# UNDERSTANDING AND MEETING THE NEEDS OF RESIDENTS IN LONG-TERM CARE FACILITIES: A MULTIDISCIPLINARY PERSPECTIVE

**DOI:** 10.1093/geroni/igz038.904

**Published:** 2019-11-08

**Authors:** Barbara Hanratty, Karen Spilsbury

**Affiliations:** 1 Newcastle University, Newcastle upon Tyne, United Kingdom; 2 University of Leeds, Leeds, United Kingdom

## Abstract

Long-term care facilities play a vital role in the care of older people. Across the world, service providers face common challenges to the delivery of high quality care to residents. Rising levels of morbidity and dependency, recruiting and retaining a skilled workforce, and separation from mainstream services are some of the issues that make this one of the most precarious care sectors. In this symposium, we will consider the evidence underlying some of these challenges, along with current and possible future service responses. The first presentation will look at factors that increase the risk of transition to dependency in the Newcastle 85+ cohort study. This is followed by an analysis of trends over time in health, morbidity and disability in the UK care home population, drawing on data from three later life cohorts. Having considered the characteristics and needs of residents, the next presentations move onto care services. Findings will be presented from a mixed methods study on the relationship between care home staffing and quality of care, followed by a study of the organisation of primary care for long term care facilities. This session will end by looking to the future, with findings from rapid syntheses of international evidence on technology, and evaluation methods, in animated format. Together, these presentations will enhance our understanding of the relationships between the needs of residents in long-term care facilities, demands on service providers and quality of care. We aim to stimulate debate and discussion on future directions for research and practice.

